# Assessing Risk of *E. coli* Resuspension from Intertidal Estuarine Sediments: Implications for Water Quality

**DOI:** 10.3390/ijerph16183255

**Published:** 2019-09-05

**Authors:** Adam J. Wyness, David M. Paterson, James E. V. Rimmer, Emma C. Defew, Marc I. Stutter, Lisa M. Avery

**Affiliations:** 1 Sediment Ecology Research Group, Scottish Oceans Institute, School of Biology, University of St Andrews, East Sands, St. Andrews KY16 8LB, UK (D.M.P.) (J.E.V.R.) (E.C.D.); 2 Environmental and Biological Sciences Group; The James Hutton Institute, Craigiebuckler, Aberdeen AB15 8QH, UK (M.I.S.) (L.M.A.); 3 Coastal Research Group, Department of Zoology and Entomology, Rhodes University, Grahamstown 6139, South Africa; 4 Lancaster Environment Centre; Lancaster University, Bailrigg, Lancashire LA14YQ, UK

**Keywords:** estuarine sediment, intertidal, cohesive sediment, sediment stability, erosion, faecal contamination, *E. coli*, faecal indicator organism (FIO), bathing waters, water quality

## Abstract

Estuarine sediments are a reservoir for faecal bacteria, such as *E. coli*, where they reside at greater concentrations and for longer periods than in the overlying water. Faecal bacteria in sediments do not usually pose significant risk to human health until resuspended into the water column, where transmission routes to humans are facilitated. The erosion resistance and corresponding *E. coli* loading of intertidal estuarine sediments was monitored in two Scottish estuaries to identify sediments that posed a risk of resuspending large amounts of *E. coli*. In addition, models were constructed in an attempt to identify sediment characteristics leading to higher erosion resistance. Sediments that exhibited low erosion resistance and a high *E. coli* loading occurred in the upper- and mid-reaches of the estuaries where sediments had higher organic content and smaller particle sizes, and arose predominantly during winter and autumn, with some incidences during summer. Models using sediment characteristics explained 57.2% and 35.7% of sediment shear strength and surface stability variance respectively, with organic matter content and season being important factors for both. However large proportions of the variance remained unexplained. Sediments that posed a risk of resuspending high amounts of faecal bacteria could be characterised by season and sediment type, and this should be considered in the future modelling of bathing water quality.

## 1. Introduction

Estuarine sediments act as a reservoir for faecal bacteria that have been transported to the environment from both point and diffuse sources throughout the watershed. In the water column faecal bacteria can occur as free cells but also adhered to suspended particles, which results in the accumulation of faecal bacteria in sediments when particles are deposited [1]. The sediment environment improves the survival rates of faecal indicator organisms (FIOs) compared to overlying water by providing protection from biotic and abiotic stressors [2,3,4], and FIO survival has been observed for up to several months [5,6]. Faecal bacteria in estuarine sediments do not usually pose significant risk to human health until they are resuspended into the water column, when sediments themselves become a source of contamination. This can be through (a) mass erosion where sediment beds are eroded during storm conditions, (b) small scale erosion from tidal or current shear forces entraining the uppermost sediment layers [7,8] or (c) organismal reworking where infauna such as the mud shrimp, *Corophium volutator,* resuspend sediment as part of their bioturbatory behaviour [9]. Once in the water column, tidal currents may transport suspended bacteria away from contaminated sites to areas where the likelihood of human contact is increased, such as bathing waters and sandy beaches. The driving factors for the abundance of FIOs in estuarine sediments are well documented [2,4,10,11,12], and these data are now being combined with hydrological catchment models to predict resuspension of FIOs. However, predicting the resuspension of estuarine sediments under certain rainfall and flow conditions is problematic since the erosion resistance of sediments is notoriously site-specific and ‘patchy’ [8,13,14]. There are many factors influencing erosion resistance including the hydrodynamic setting and physical sediment characteristics [14,15,16]. In addition, organic material and other biological factors such as extracellular polymeric substances (EPS) content, infaunal activity and biofilm formation can have both stabilising and destabilising effects [17]. 

The measurement of sediment properties related to stability is complex, and two properties are often measured: shear strength and surface stability [14,18]. Sediment shear strength is a measure of the “internal strength” of the sediment as expressed by its resistance to torque, and is used as an indicator of its susceptibility to mass erosion. Shear strength has been shown to increase with an increase in sediment packing (bulk density) and a reduction in water content [14,19]. The surface stability is the resistance of sediments to surface shear stress which is regarded to be the dominant form of sediment transport within tidal waters [20] and is affected by physical factors such as particle size distribution, but also biological mediation such as biofilm development [21] and bioturbation [22].

Estuarine sediments can be broadly divided into two groups; cohesive and non-cohesive sediments. In sediments consisting mostly of particle sizes >63 µm, particles act independently and particle size becomes more critical than inter-particle cohesion. On the other hand, in sediments with particle sizes <63 µm stability increases with smaller particle sizes as surface attraction (cohesion) between particles becomes important. In mixed sediments (cohesive and non-cohesive) if there is a >10% fraction of fine particles (<63 µm), sediments start to display cohesive properties resulting in higher sediment stability [23]. 

In addition to physical characteristics, there is a large influence on sediment stability by actions of microorganisms [24,25,26], diatoms [20,27], invertebrates [28] and macrofauna [17,22]. Perhaps the most widely researched biotic factor affecting the stability of sediments is that of the EPS constituent of microphytobenthic biofilms [17,26,29,30]. The EPS form a cohesive network by covering particles and spanning gaps between them [17], physically binding sediments and increasing the erosion threshold [20]. Microalgae are the primary source of EPS in intertidal mudflats [31], primarily exuding carbohydrates, whereas bacteria generally produce more protein-rich exudates [32]. EPS constituents can be quantified separately, but also forms a major constituent of the total organic content. 

The main aim of this study was to ascertain spatial and temporal trends in the occurrence of intertidal sediments that posed a high risk of resuspending large numbers of faecal bacteria. This was explored by locating sediments that contained high concentrations of faecal bacteria and exhibited low erosion thresholds, leading to a greater risk of flux of faecal bacterial to overlying waters. The data for this study has been retrieved from a larger dataset alongside many other measurements [11], consequently a secondary aim of this study was to model that the sediment characteristics that describe sediment stability and sediment shear strength in order to inform on sediment properties contributing to change in erosion threshold.

## 2. Materials and Methods

### 2.1. Sampling Sites 

**Figure 1:**
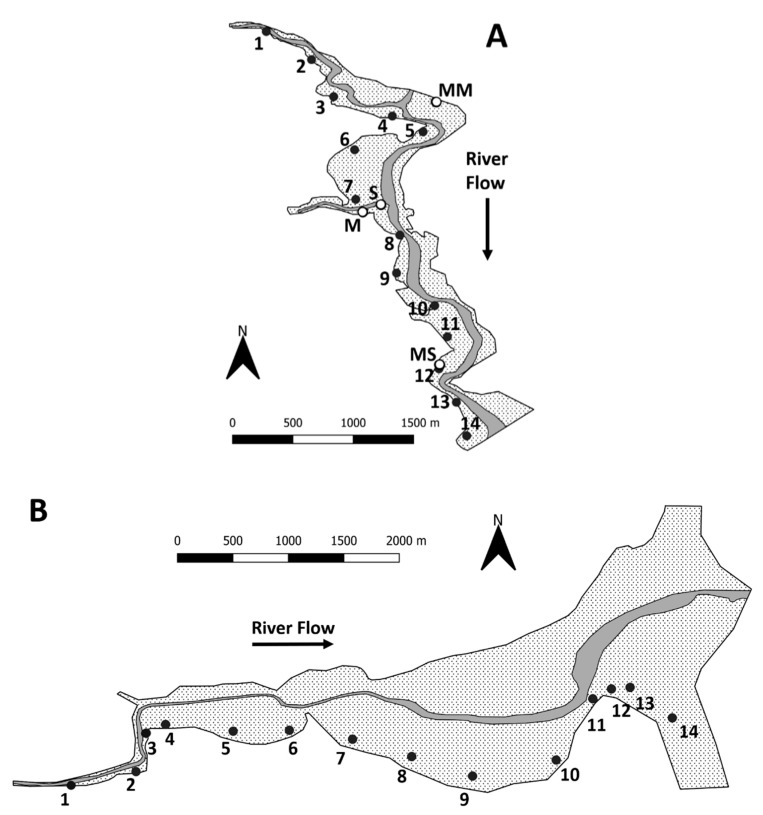
Sample collection sites on the Ythan (**A**) and Eden (**B**) estuaries. For (**A**), monthly sampling campaign sites: mud (M), mixed mud (MM), mixed sand (MS) and sand (S). For (**A**,**B**), numbers indicate transect sampling sites, dark grey represents the river channel at low tide and the dotted area indicates intertidal mudflat. Figure reprinted from *Science of the Total Environment*, Vol. 661 (15), Wyness et al. Factors affecting the spatial and temporal distribution of *E. coli* in intertidal estuarine sediments, pp. 155–167, Copyright 2019, with permission from Elsevier.

The field sampling regime had been previously described by Wyness et al. [11]. An intensive monthly sampling campaign was conducted on the Ythan estuary (Aberdeenshire, Scotland. Lat. 57.343227, Lon.-2.000371) where monthly samples were taken at 4 sites: mud (M), mixed mud (MM), mixed sand (MS) and sand (S) (Figure 1A). Seasonal transect sampling campaigns were also conducted on the Ythan and Eden estuaries (Fife, Scotland, Lat. 56.373028, Lon.-2.835975) where 14 sites were sampled, with site one at the head of the estuary, and site 14 at the mouth (Figure 1A,B). 

### 2.2. E. coli Enumeration

*E. coli* was enumerated as previously described in Wyness et al. [11]. Briefly, plastic disposable syringes (Fisher Scientific, Loughborough, UK) [33] were used to take three adjacent sediment cores to a depth of 1 cm which were combined and weighed into 100 mL sterile vessels. Seventy mL of sterile phosphate buffered solution (PBS) was added and the vessel reciprocally shaken at 30 cm and 120 rpm for 1 min and left to settle for 1 h. Thirty ml of supernatant was removed and combined with 70 mL PBS and one pack of IDEXX Colilert-18 reagent (IDEXX Laboratories, Westbrook, ME, USA) added. The manufacturer’s protocol for IDEXX Quanti-Tray/2000 was then followed and the results calculated to most probable number colony forming units (CFU) 100 g dry·wt^−1^ sediment. This method enumerates *E. coli* incorporated in surface biofilms, adhered to sub-surface sediment particles and also those not associated with particles and present in the sediment pore-water.

### 2.3. Erosion Potential of Sediments

Sediment surface stability was measured using the cohesive strength meter (CSM). The CSM determines the threshold for surface erosion using a variable jet pulse of water aimed at the sediment surface through a small water chamber [20,34]. The resistance of the bed to erosion from this jet of water impacting the sediment surface is determined by quantifying the amount of sediment resuspended by each jet by measuring the light attenuation across the chamber. Four replicate CSM measurements were taken at each individual sampling site using the most sensitive CSM programme, ‘Fine 1’. Several of the coarse-grained sand sediments were too porous to enable water retention inside the CSM chamber, so recordings were not taken. Results were reported as the stagnation pressure (Nm^−2^) of the water jet at the moment of significant surface erosion. Sediment shear strength was measured using a handheld shear vane (Geonor, Augusta, NJ, USA) using the 50 mm × 12 mm vane, therefore measuring torsion resistance from 0–50 mm depth. Four replicate measurements were taken at each site, and results recorded as kPa. 

In order to give context to the sediment characteristics that lead to higher and lower erodibility, a series of ordinary least squares general linear models was constructed using a suite of sediment characteristics. The maximal model was: season (factorial; spring—April 15th to June 30th, summer—July 1st to September 15th, autumn—September 16th to November 15th, winter—November 16th to April 14th), organic matter content (% wt), water content (% wt), bulk density (g·cm^3^), fine particles (% <63 µm), median particle diameter (µm), volume weighted mean particle diameter (μm), interstitial water pH and salinity (PSU), colloidal carbohydrates (μg·g^−1^), colloidal proteins (μg·g^−1^), tidal amplitude (m), air temperature (minimum at ground level, minimum and maximum (°C)) and precipitation for the previous day to the sampling event, the previous 2 days cumulatively and the previous 5 days cumulatively (mm).

Models were constructed using a bottom-up approach in which a single predictor was added in a stepwise fashion, with a revaluation of model quality made at each step. Model quality was assessed using AICc, (corrected Akaike’s information criterion), in place of AIC due to the relatively small sample size [35]. Quality was also assessed by adjusted R-squared and model overall significance. AICc was also used to check for higher quality nested models as predictors were added.

Diagnostic plots of residual distributions were consulted to ensure model assumptions were met, and transformations of the stability responses (shear vane and CSM) were made as appropriate. Multicollinearity was avoided by not fitting together predictors expected a priori to be highly collinear, such as the different temperature metrics, as well as by the consultation of each predictor’s variable inflation factor. Where high collinearity occurred, the poorer predictor was excluded. Analysis and model building were carried out using R 3.5.1 (R Foundation for Statistical Computing, Vienna, Austria) [36].

## 3. Results

### 3.1. Spatio-Temporal Variability in E. coli Resuspension Potential in the Ythan Estuary

Sediments with a shear strength of <5 kPa or a surface stability of <20 Nm^−2^, and containing an abundance of *E. coli* greater than 3 log^10^ CFU 100g dry·wt^−1^ were considered to be at a high risk of resuspending a significant amount of faecal bacteria into the water column.

Within the monthly sampling campaign, sediment shear strength was greatest at the mixed sand (MS) site followed by mixed mud (MM) > sand (S) > mud (M) (mean ± SE: MS 13.12 ± 0.54, MM 8.38 ± 0.26, S 5.00 ± 0.27, M 3.55 ± 0.15) all of which were significantly different from each other (ANOVA; site; F (3, 290) = 201.8, *p* < 0.001). Shear strength was greatest during spring and autumn followed by summer > winter (mean ± SE: spring 8.63 ± 6.01, autumn 8.43 ± 0.66, summer 7.14 ± 0.41, winter 6.36 ± 0.37) (ANOVA; season; F (3, 290) = 12.5, *p* < 0.001). The interaction between site and season was significant, however weaker than the 1-way effects (ANOVA; site x season; F (9, 290) = 7.3, *p* < 0.001). Shear strength within M and S did not vary widely throughout the year (Supplementary Figure S1), but there was a general decrease from summer to winter. At MM, shear strength was significantly greater during autumn than other seasons, during which it did not significantly differ. Shear strength at MS was variable between seasons, with the greatest shear strength observed during spring. 

Sediment stability was greater at MS and MM than M (mean ± SE: MS 4.39 ± 0.29, MM 3.86 ± 0.26, M 2.95 ± 0.19) (ANOVA; site; F (2, 160) = 14.6, *p* < 0.001). No sediment stability data was recorded at S because of the porous nature of the sediment. Data was absent for MM during summer because of seasonal growth of macroalgae at this site that obscured the sediment. Sediment stability was greater during spring than winter and autumn, and stability during summer was not significantly different than any other season (mean ± SE: spring 4.40 ± 0.22, summer 4.15 ± 0.34, winter 3.29 ± 0.27, autumn 2.99 ± 0.31) (ANOVA; season; F (3, 160) = 7.3, *p* < 0.001). Sediment stability at M was greater during spring and summer than autumn and at MM was higher during spring and autumn than winter. Sediment stability at MS was greater during winter than all other sediment–season combinations except MM during spring (ANOVA; site x season; F (5, 160) = 11.5, *p* < 0.001) (Supplementary Figure S2).

**Figure 2:**
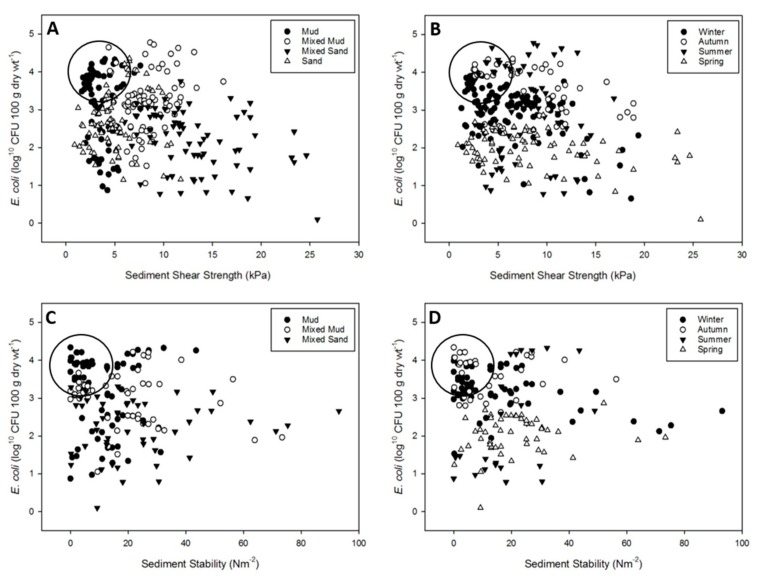
Scatterplot of observed *E. coli* abundance in colony forming units (CFU) vs. erosion resistance for the monthly sampling on the Ythan estuary dataset. Plots (**A**) and (**C**), solid circles: mud; hollow circles: mixed mud; solid triangles: mixed sand; hollow triangles: sand. Plots (**B**) and (**D**), solid circles: spring; hollow circles: summer; solid triangles: autumn; hollow triangles: winter. Circles highlight samples with high *E. coli* abundance and low erosion resistance.

Sediments containing high *E. coli* abundance and low erosion resistance were most commonly observed in M during winter and autumn (Figure 2). Sediments with low shear strength and high *E. coli* abundance were mostly in M during winter and autumn, with some MM during summer (Figure 2A,B). Low sediment stability was again observed in M and MM sediments, and exclusively during winter and autumn (Figure 2C,D). 

### 3.2. Resuspension Potential of E. coli: Seasonal Transects at Two Estuaries

**Figure 3:**
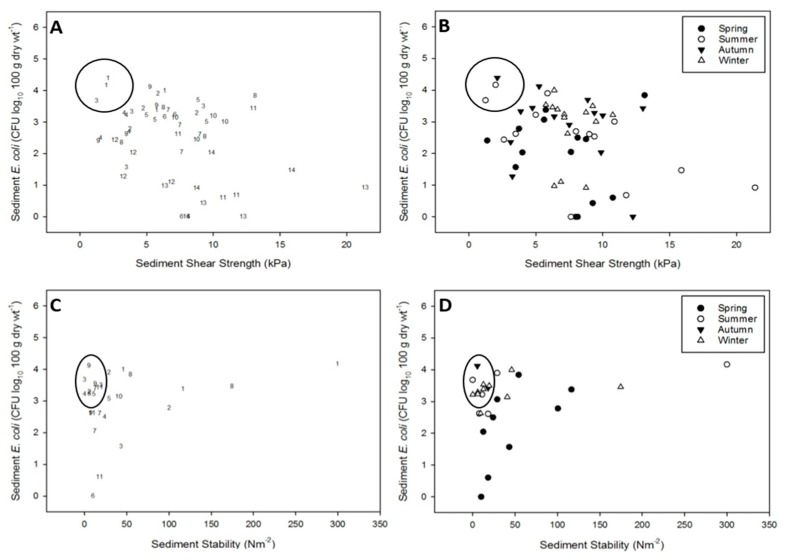
Scatterplots of observed *E. coli* abundance (CFU) vs. erosion resistance for the Ythan estuary transect dataset. Plots (**A**) and (**C**), numbers denote sampling position within the estuary with 1 at the head of the estuary and 14 at the mouth. Plots (**B**) and (**D**), solid circles: spring; hollow circles: summer; solid triangles: autumn; hollow triangles: winter. Circles highlight samples with high *E. coli* abundance and low erosion resistance.

**Figure 4:**
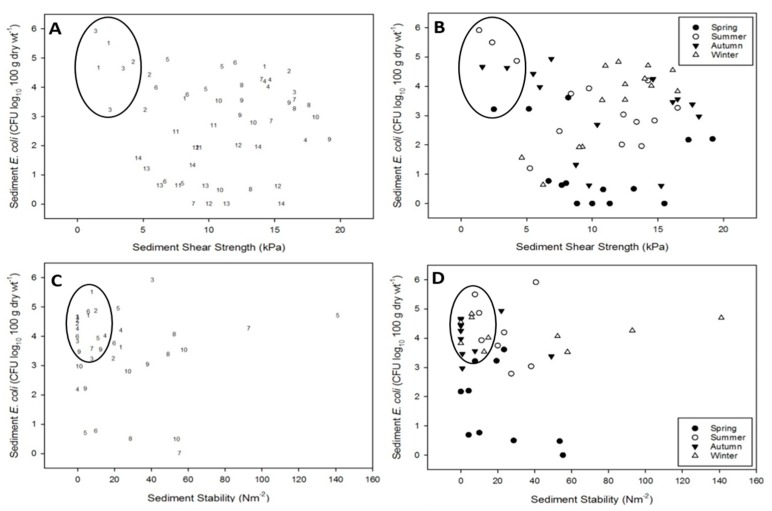
Scatterplots of observed *E. coli* abundance (CFU) vs. erosion resistance for the Eden estuary transect dataset. Plots (**A**) and (**C**), numbers denote sampling position within the estuary with 1 at the head of the estuary and 14 at the mouth. Plots (**B**) and (**D**), solid circles: spring; hollow circles: summer; solid triangles: autumn; hollow triangles: winter. Circles highlight samples with high *E. coli* abundance and low erosion resistance.

Generally, *E. coli* abundance was highest in both estuaries at the head of the estuary, with numbers gradually decreasing towards the mouth of the estuary. The total load of *E. coli* in each estuary was highest in the summer, followed by autumn, winter then spring. Low shear strength combined with high *E. coli* abundance was observed at upper-estuary sediments (sites 1–3) in both estuaries, and occurred predominantly during summer and autumn (Figure 3A,B and Figure 4A,B). 

Sediments with low stability were more widely distributed throughout the upper- to mid-reaches of both estuaries, and occurred during summer, autumn and winter (Figure 3C,D and Figure 4C,D). Sediments during spring typically demonstrated low sediment stability, but *E. coli* abundance was generally low.

### 3.3. Modelling of Erosion Thresholds

The best model for explaining sediment shear strength indicated that five predictors explained 57.2% of the variance (F (8, 255) = 44.94, *p* < 0.010). After a log_e_ transformation of shear strength, it was found that stability significantly increased with organic matter content as median particle diameter increased (and vice versa), and also significantly increased as bulk density increased. In contrast, increasing alkalinity significantly decreased stability. Spring, summer and winter showed reduced shear strength relative to autumn (Table 1). 

The best model for sediment stability explained less of the variation than that for the shear strength. Five predictors explained 35.7% of the variance (F (8, 137) = 11.05, *p* < 0.001) (Table 2). After a cube root transformation of stability, it was found that increased colloidal carbohydrates significantly increased stability, whereas percentage organics was found to significantly reduce stability. All months showed increased stability relative to autumn, but this increase was not significant for winter. Stability significantly increased with tidal amplitude, conditional on an increase in 5-day precipitation.

There was a strong influence of season on models for both sediment shear strength and sediment stability. The model for sediment shear strength featured the physical sediment characteristics of mean particle diameter and bulk density in addition to organic matter content and pH, whereas the model for sediment stability featured colloidal carbohydrates, 5-day cumulative precipitation and tidal amplitude in addition to organic matter content.

## 4. Discussion

Sediment erosion thresholds observed here are consistent with values obtained in previous studies [8,14,37] suggesting the results here may be widely applicable. There was no significant correlation between sediment shear strength and stability over the two estuary transects, or within the monthly sampled sediment types except mixed sand (data not shown). This may be expected because, as discussed in the introduction, different variables affect sediment shear strength, and sediment stability.

Current predictions of bathing water quality advisories in Scotland are created using historical correlations between bacteriological data, and rainfall (up to 72 hours before, and 12 hour forward prediction) and river flow data [38,39]. This generated a 69% success rate of correctly predicting water quality advisories, and 99% success rate of correctly predicting, or predicting a higher level of contamination than occurred at 23 locations in 2014 [40]. It is unclear however whether this success rate will be similar for areas susceptible to contamination arising from sediment resuspension such as those within and near to estuaries, which are still used heavily by the public. 

The abundance of *E. coli* and sediment erosion thresholds displayed little correlation, however, specific sediment types and times of year that posed a higher risk of suspending high levels of *E. coli* into the water column were noticeably identified. Many of these ‘high risk’ sediments occurred during summer and autumn, which overlaps with the Scottish Environment Protection Agency monitored bathing season in Britain (mid-May–mid-September). During these periods, it may be beneficial to monitor erosion thresholds of sediments to identify where there is an increased risk of the resuspension of large numbers of FIOs from estuarine sediments. Assessment of risk may be performed using *in situ E. coli* abundances, or inferred using general sediment characteristics [10,11].

Sediment-borne FIO resuspension is also pertinent to aquaculture and its transmission routes to humans. For example, shellfish flesh is known to be an environmental reservoir of FIOs [41] therefore presenting a possible hazard to human health when consumed. Clements et al. [42] observed no correlation between the total coliform and *E. coli* abundance in mussel (*Mytilus edulis*) tissues and spatially corresponding surface sediments, therefore direct measurement or prediction of *E. coli* abundance in sediments directly underlying shellfish beds are unlikely to be of use. However, knowledge of the potential for upstream or estuarine sediments to resuspend and release FIOs into the water column which then may accumulate in shellfish tissue through filter feeding is of importance to determining and identifying the causes of bioaccumulation of FIOs in shellfish. 

Models predicting concentrations of waterborne *E. coli* do include sub-models accounting for resuspension of *E. coli* from underlying sediments [2,43,44]. Current models utilise set parameters to simulate a critical shear stress that, when exceeded, account for the resuspension of bacteria from sediments; the value used for this parameter varies between 0.02 (for fine coastal bay sediments) to 1.7 Nm^−2^ (streambeds) [45,46]. Yang et al. (2008) introduced a differential critical shear stress for cohesive and non-cohesive types of estuarine sediment to improve model accuracy for predicting enteric bacterial resuspension, however the critical shear stress for sediment erosion is based upon particle size without consideration of sediment biogeochemistry or biogenic factors [47]. 

The models predicting both types of erosion resistance of sediments do not explain very large proportions of the variation in the dataset (R^2^: 0.57 and 0.36 for shear strength and stability respectively). This was possibly a result of only physical and biochemical parameters being considered. Biogenic impacts on sediment stability can be sizeable, for example the influence of *Nereis diversicolor*, a burrowing polychaete which has been reported to both stabilize and destabilize sediments in different environments [48]. Many examinations of the biogenic impacts on sediment stability have been made [17,49], but the accurate determination and modelling of these effects remain hugely challenging due to variation in the relative diversity, abundance and activity of fauna present in intertidal systems, and their wide-ranging and context-dependent burrowing and feeding regimes. 

Seasonality strongly influenced both measures of erosion resistance. This is perhaps not surprising as it is indicative of many interacting physical, biological and chemical changes that were otherwise not measured, for example, sunlight intensity and sediment exposure time, abundance and activity of both stabilizing and destabilizing macrofauna and change in river water ionic matrix and organic matter constituents. A greater abundance of filamentous macro-algae has been regularly reported during late summer and early autumn compared to other seasons, especially in the upper reaches of the Ythan estuary [50]. Macro-algal filaments can penetrate several centimetres into the sediment and form a dense matrix which could have contributed to the increase in sediment shear strength during autumn. *Corophium volutator* is a very active borrowing amphipod that has been demonstrated in laboratory studies to increase sediment shear strength with greater densities [51]. *C. volutator* populations peak in the Ythan estuary between July and September [52], suggesting this could have contributed to the seasonal variation of sediment shear strength. There was a strong effect size of the interaction between organic content and particle size, signifying that this adhesion of particles had a greater effect on sediment shear strength when particle size was larger. It may be logical to suppose this, as sediments with larger particle sizes have a greater porosity and therefore a lower water content. This results in an increased shear strength, which then increases with the addition of adhesive organic matter. Organic matter alone did not have as large an effect, as organic content is often positively correlated with water content in intertidal sediments [11], which adversely affects erosion thresholds. The shear strength model also featured bulk density which is indicative of the packing density of sediments, which is to be expected [14,19]. Organic matter in sediments bind particles together, developing larger aggregates and adhering particles together [15]. 

Colloidal carbohydrates (a measure of EPS biofilm content), and bulk organic matter content were significant model terms predicting sediment stability. As described earlier, EPS presence has a strong influence on sediment stability due to the increased cohesion between sediment particles [20]. Likewise, organic matter content has been previously reported to increase sediment stability by increasing particle adhesion [15] and by fibrous organic material providing a structured physical barrier on the sediment surface [53]. Such decomposing fibrous structure was observed in the upper reaches of the Ythan estuary and resulted in the highest sediment stability determined in this study. Caution must be taken in drawing strong conclusions from the relatively small standardised coefficients, however, larger tidal amplitude and higher cumulative precipitation were both related to an increase in sediment stability, and this may be a consequence of longer sediment exposure time leading to higher surface evaporation and a ‘hardening’ of the surface layers as described by Amos et al. [37]. 

Rainfall is typically associated with a decrease in sediment stability by physically disturbing sediment or dissolving EPS constituents [54]. However it may have had a positive effect on the quantity or quality of EPS biofilms by introducing nutrients via river exports, and redistributing nutrients within estuaries [55], or inducing a stress response (increased polymer production) in microphytobenthos to physical disturbance or increased shear force as a result of a larger water discharge through the estuary. The additional interaction of increased rainfall and larger tidal amplitudes requires further investigation. 

## 5. Conclusions

The data presented here demonstrate there was little correlation between sediment erosion thresholds and FIO abundance. However, there was spatial and temporal structure to the sediments that contained a high FIO abundance and exhibited low erosion thresholds, with slightly different trends for each erosion measure. These sediments predominantly occurred during autumn and winter, and were muddy sediments (high organic content and small particle size), usually located in the upper sections of the estuaries. There was sufficient structure to these trends that when incorporating erosion potential thresholds into models predicting resuspension of FIOs into the estuarine water column, both large-scale (shear strength) and small-scale (stability) resuspension parameters should be considered. The secondary aim in this study was to use sediment characteristics to explain the variability of erosion thresholds. The models were moderately and poorly successful for sediment shear strength and surface stability respectively, and more work is necessary to elucidate the drivers of erosion thresholds. In conclusion, it has been demonstrated that sediments that pose a risk of resuspending high amounts of faecal bacteria can be characterised by season and sediment type, and this should be considered in the future modelling of bathing water quality. 

## Figures and Tables

**Table 1:** Coefficients and centred, standardised coefficients for the best model predicting sediment shear strength. Adjusted R2: 0.532, F (7, 256): 43.63, *p* < 0.001. *p*-values are given for the standardised coefficients.

Predictor	Coefficient	Standardised Coefficient	*p*-Value
Intercept	8.625	2.978	<0.001
Organic matter content	−0.549	0.230	<0.050
Season: spring	−0.534	−0.534	<0.001
Season: summer	−0.629	−0.630	<0.001
Season: winter	−0.398	−0.398	<0.001
Median particle diameter	−0.006	0.548	<0.001
pH	−0.740	−0.196	<0.001
Bulk density	−0.245	0.065	<0.050
Organic matter content × Median particle diameter	0.004	0.929	<0.001

**Table 2:** Coefficients and centred, standardised coefficients for the best model predicting sediment stability. Adjusted R2: 0.357, F (8, 137): 11.05, *p* < 0.001. *p*-values are given for the standardised coefficients.

Predictor	Coefficient	Standardised Coefficient	*p*-Value
Intercept	2.859	1.923	<0.001
Organic matter content	−0.202	−0.312	<0.001
Season: spring	0.653	0.653	<0.001
Season: summer	0.683	0.683	<0.001
Season: winter	0.181	0.181	0.341
Colloidal carbohydrates	0.002	0.253	<0.001
5-day cumulative precipitation	−0.098	0.124	0.117
Tidal amplitude	−0.338	0.161	0.063
5-day cumulative precipitation × Tidal amplitude	0.042	0.248	<0.001

## Supplementary Materials

The following are available online at https://www.mdpi.com/1660-4601/16/18/3255/s1, Figure S1: means plot from the two-way ANOVA (site × season) for sediment shear strength at four sediment types in the Ythan estuary. Error bars denote standard error from the mean. Solid fill bars: mud; light-grey dashed bars: mixed mud; dark-grey dashed bars: mixed sand; hatched bars: sand. Sediment stability measures are absent in mixed mud during summer, and in sand during all seasons. Significant differences between treatment means are those larger than the max-min LSD value. Figure S2: means plot from the two-way ANOVA (site × season) for sediment surface stability at four sediment types in the Ythan estuary. Error bars denote standard error from the mean. Solid fill bars: mud; light-grey dashed bars: mixed mud; dark-grey dashed bars: mixed sand; hatched bars: sand. Sediment stability measures are absent in mixed mud during summer, and sand during all seasons. Significant differences between treatment means are those larger than the max-min LSD value.
