# Supplementary material for: Assessing Risk of E. coli Resuspension from Intertidal Estuarine Sediments: Implications for Water Quality
Source: Int J Environ Res Public Health. 2019 Sep 5;16(18):3255. doi: 10.3390/ijerph16183255 (PMC6765901; doi:10.3390/ijerph16183255)
Supplement: Supplementary file 1 [file ijerph-16-03255-s001.pdf]

International Journal of
Environmental Research and Public Health

Supplementary Materials: Assessing Risk of *E. coli* Resuspension from Intertidal Estuarine Sediments: Implications for Water Quality

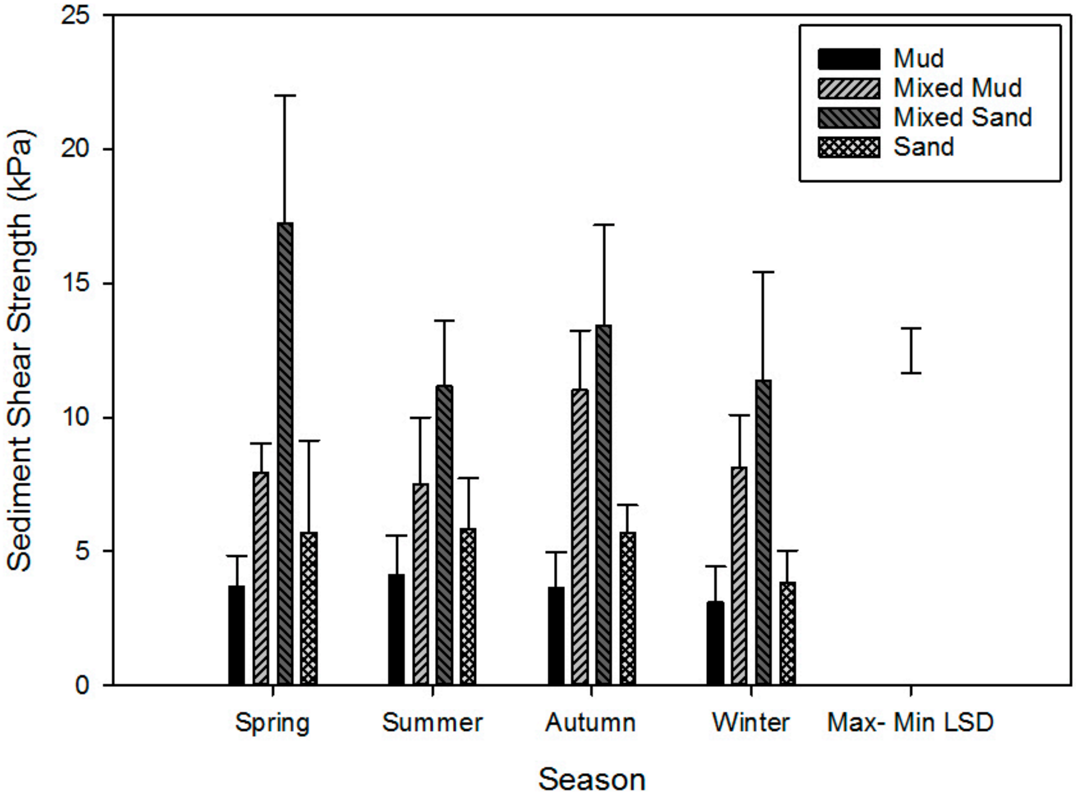

Figure S1. Means plot from the two-way ANOVA (site × season) for sediment shear strength at four sediment types in the Ythan estuary. Error bars denote standard error from the mean. Solid fill bars: mud; light-grey dashed bars: mixed mud; dark-grey dashed bars: mixed sand; hatched bars: sand. Sediment stability measures are absent in mixed mud during summer, and sand during all seasons. Significant differences between treatment means are those larger than the max-min LSD value.

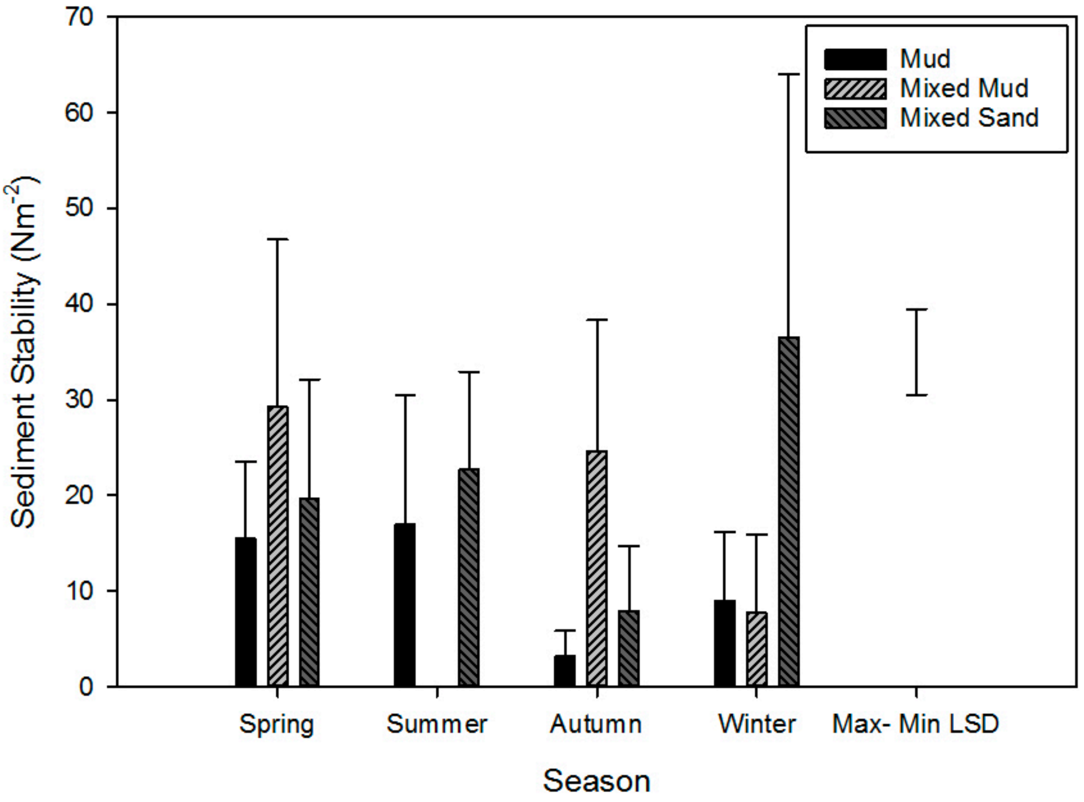

Figure S2. Means plot from the two-way ANOVA (site × season) for sediment surface stability at four sediment types in the Ythan estuary. Error bars denote standard error from the mean. Solid fill bars: mud; light-grey dashed bars: mixed mud; dark-grey dashed bars: mixed sand; hatched bars: sand. Sediment stability measures are absent in mixed mud during summer, and sand during all seasons. Significant differences between treatment means are those larger than the max-min LSD value.

© 2019 by the authors. Submitted for possible open access publication under the terms and conditions of the Creative Commons Attribution (CC BY) license (http://creativecommons.org/licenses/by/4.0/).
